# The Future of Training and Practice in Neuromodulation: An Interventional Psychiatry Perspective

**DOI:** 10.3389/fpsyt.2021.734487

**Published:** 2021-08-27

**Authors:** Nicholas T. Trapp, Nolan R. Williams

**Affiliations:** ^1^Department of Psychiatry and Behavioral Sciences, Stanford University, Stanford, CA, United States; ^2^Department of Psychiatry, University of Iowa, Iowa City, IA, United States

**Keywords:** interventional psychiatry, neuromodulation, repetitive transcranial magnetic stimulation, electroconvulsive therapy, deep brain stimulation, vagus nerve stimulation, training, fellowship

## Introduction

Psychiatrists have been proceduralists dating back almost 100 years, nearly to psychiatry's inception as a medical specialty. Electroconvulsive therapy (ECT) was developed as a procedure in the 1930s and refined over time, with impressive efficacy and response rates (1). However, due to a lack of understanding of the pathophysiology of many psychiatric conditions, combined with disastrous outcomes from premature adoption of other procedural treatments such as transorbital lobotomy (2), procedural options for psychiatric treatment have been limited. In recent years this landscape is changing, again introducing the potential for procedural therapies in neuropsychiatric disease management.

The subspecialty focused on procedure-based psychiatric care, specifically utilization of neurotechnologies to treat psychiatric disorders has become known as “interventional psychiatry.” Interventional psychiatry frequently employs treatments under the umbrella of “psychiatric neuromodulation,” which we define as the collection of nervous system stimulation therapies focused on modulating dysfunctional brain circuitry for therapeutic benefit, including use of electrical, magnetic, ultrasonic, and photic stimulation (see Table 1 for examples). As practically applied, interventional psychiatry also often incorporates procedure-based pharmacologic interventions such as ketamine infusion therapy and psychedelic therapies, which similarly involve a medical intervention applied to a treatment-refractory patient population and require familiarity with procedural consent and monitoring. Here we outline the state of the field, as well as implications for training and the role of the interventional psychiatrist in the treatment team.

**Table 1:** Proposed competency areas for trans-disciplinary fellowship in interventional psychiatry or psychiatric neuromodulation.

**CORE COMPETENCIES:**
***Invasive neuromodulation***
- Indications, Evaluation, Procedure, Periprocedural Care (DBS, VNS, RNS, SCS, epCS) - In depth knowledge of treatment approaches, pharmacologic and psychotherapeutic treatment alternatives, risks/benefits/side effects of each procedure - Demonstrated skill and knowledge for seeking and obtaining patient consent, answering patient and family questions about treatment options - Demonstrated proficiency in device management including initial programming, impedance checks, troubleshooting device issues, intraoperative neuropsychiatric assessments of patients during awake DBS procedures - Knowledge of how and when to interface with neurology, neurosurgery, and device manufacturers - Troubleshooting treatment failures or lack of response to standard settings - Communication of expectations pre-procedure, patient education, follow-up care, maintenance, and augmentation strategies
***Non-invasive neuromodulation***
- **Modalities:** ***Electrical** = tES, ECT, TNS, PNS; **Magnetic** = TMS, MST; **Pharmacologic** = ketamine, esketamine, psychedelics; **Ultrasonic** = FUS; **Photic** = photobiomodulation, gamma light therapy, optogenetics; **Haptic** = micromotor stimulation*- Indications, Evaluation, Procedure, Periprocedural Care - In depth knowledge of treatment approaches, pharmacologic and psychotherapeutic treatment alternatives, risks/benefits/side effects of each procedure - Demonstrated skill and knowledge for seeking and obtaining patient consent, answering patient and family questions about treatment options - Demonstrated skill in administration of each treatment modality, troubleshooting issues and managing side effects and complications - Troubleshooting treatment failures and lack of response to standard protocols or settings - Skill in procedure-specific techniques such as reading/interpreting EEG in ECT treatments, achieving motor threshold in TMS procedures, delivering provocation in TMS for OCD - Knowledge of techniques currently used off-label or without an FDA-approved indication, including potential research applications and future clinical potential - Communication of expectations pre-procedure, patient education, follow-up care, maintenance and augmentation strategies
***Neuroimaging**-diagnostic and functional*
- Demonstrated proficiency in localization of neuroanatomical structures and white matter tracts, especially those of relevance for neuromodulation targeting and therapy
***Neuropsychiatric evaluations** with a focus on treatment refractory OCD, depression, and other conditions amenable to neuromodulation* * intervention*
- Knowledge of common psychiatric symptom rating scales to quantify disease characteristics and treatment response - Knowledge of proposed neurocircuitry involved in neuropsychiatric diseases and proposed therapeutic options
***Cross-disciplinary neuropsychiatric evaluations** for patients with conditions amenable to neuromodulation intervention*
- Knowledge of indications/contraindications for neuromodulation, necessary work-up and evaluation, treatment alternatives, differential diagnostic considerations
**Subspecialty evaluations may include**
- *Movement Disorder Evaluations* with a focus on Tourette's, Parkinson's Disease, Essential Tremor, Dystonia
- *Epilepsy Evaluations, Epilepsy Monitoring Unit Patient Management* with a focus on reading EEG or qEEG, cortical mapping procedures in EMU - *Pain Evaluations*- *Neurorehabilitation Evaluations*- *Neurosurgical Evaluations* with a focus on medical contraindications to surgical intervention, surgical approaches and ability to weigh risks and benefits of different surgical interventions, necessary preoperative and perioperative work-up and management, post-operative care and device/wound management
***Professionalism and communication skills** especially with multidisciplinary care and interfacing with other specialties*
***Active learning** demonstrated by contributions to the field such as scientific publication, quality improvement project, reading and interpreting new relevant journal articles*
***Cross-disciplinary fellowship options** with potential for disease-specific procedural focus*
- Psychiatry track with emphasis on TMS, ECT, VNS, ketamine - Neurology track emphasizing DBS, FUS, VNS - Pain management track emphasizing SCS, tES, TMS, ketamine - Neurorehab/PM&R track emphasizing TMS, intrathecal baclofen, SCS

*DBS, deep brain stimulation; VNS, vagus nerve stimulation; RNS, responsive neurostimulation; SCS, spinal cord stimulation; epCS, epidural cortical stimulation; tES, transcranial electrical stimulation; ECT, electroconvulsive therapy; TNS, trigeminal nerve stimulation; PNS, peripheral nerve stimulation; TMS, transcranial magnetic stimulation; MST, magnetic seizure therapy; FUS, focused ultrasound*.

## The Expansion of Neuromodulation and Advent of Interventional Psychiatry

There has been an explosion of technology in recent years capable of interfacing with the nervous system. Much of this technology focuses on treating conditions with some evidence for circuit-based or brain network pathology, such as depression, obsessive-compulsive disorder, chronic pain, movement disorders, substance abuse, and epilepsy seizure propagation (3–8). Modulation of brain regions implicated in these circuits or networks has demonstrated therapeutic efficacy, resulting in a multitude of clinical applications across a range of medical disciplines including psychiatry, neurology, neurosurgery, and pain management (9).

The field of interventional psychiatry was conceptualized in its modern form <10 years ago (10, 11). Around that time, a whole host of novel procedural treatments and devices were entering the psychiatric clinician's armamentarium (12–14). Vagus nerve stimulation (VNS) was FDA-approved for adjunctive treatment of depression (2005), transcranial magnetic stimulation (TMS) and deep TMS were FDA-cleared for treatment-refractory depression (2008 & 2013), and deep brain stimulation (DBS) received a humanitarian device exemption for obsessive-compulsive disorder (2009). Early evidence of the rapid efficacy of ketamine infusions for treatment-resistant depression was mounting (15) and novel convulsive therapies were in development including magnetic seizure therapy (MST) and FEAST (16, 17). However, with these new technologies arose concerns about how to disseminate newfound knowledge and ensure adequate training of clinicians on the procedural aspects of care. Interventional psychiatry arose as a notion designed to unify providers around a common craft, to foster discussions about training requirements and their incorporation into residency programs, and to gather like-minded clinicians to guide and further incorporate neurotechnologies in a rapidly expanding field.

## The State of Interventional Psychiatry Training

The rapid technological advances in neurotherapeutics over the past few decades have relegated prior psychiatric training standards as inadequate for the successful management and administration of these emerging neurotechnologies in psychiatry. Indeed, prior to the past 10–20 years, a psychiatrist's procedural training likely included exposure to electroconvulsive therapy at best, with residency training competencies focused primarily on “understanding the indications and uses” of ECT (18) with no requirement for procedural exposure. This remains a limitation of many training programs today, perpetuated by updated training milestones which make non-specific recommendations with no requirements for direct exposure or procedural training in interventional techniques (19, 20).

The level of training a physician may have prior to offering these therapies is highly variable. For ECT, most institutions or hospital systems have credentialing processes in place to ensure some level of proficiency prior to independent practice; however, there is no national standard. The situation is even less standardized for TMS, where many institutions lack a credentialing process. Procedural training experiences for clinicians may vary from a 1-h training from the device manufacturer to a week-long workshop, a month-long residency elective, or more recently, a year-long fellowship in interventional psychiatry. Although evidence linking more experience to better patient outcomes is limited, data suggests that technical aspects of these procedures, such as reliable TMS treatment targeting, are difficult even for trained technicians. Thus, more experience may lead to better technique, such as more reliable TMS treatment targeting or lower TMS motor thresholds (indicating more accurate treatment dosing) (21). Determining adequacy of training can be challenging, and standardizing this across institutions or regions presents an even greater challenge. As the subspecialty of interventional psychiatry continues to grow, more formalized training requirements may be considered to ensure proficiency of providers for optimal patient safety and treatment outcomes.

## Should Interventional Psychiatry Require Fellowship Training?

One common method to ensure adequate training and competency amongst physicians within a clinical discipline is the development of a formalized fellowship training program. Fellowships have the benefit of regulating training and monitoring a trainee's completion of various milestones or competencies, with the detriment of limiting clinician access to certain practice privileges in some scenarios, and thus limiting patient access to care. Informal or non-accredited fellowships can provide similar training experiences, but without the same standardization or regulation. Both formal and informal fellowships carry the drawback of requiring clinicians to spend additional time after residency engaged in training, thus further delaying their ability to enter independent practice.

An alternative to fellowship training would be incorporating interventional psychiatry training into residency programs (11). Although ideal in many respects, there are also many limitations. Many psychiatry training programs do not have access to interventional psychiatry equipment, technology, and expertise. Indeed, ECT is the most long-standing neuromodulation technique employed in psychiatry, and yet it lacks a foothold in many training programs. One recent study showed that only 75% of psychiatry residency training programs required residents to have clinical exposure to ECT, and only 57% had a dedicated ECT rotation (22). In addition to a lack of access, there is also a lack of time. Residents have numerous demands on their time and an ever-increasing list of competencies and learning expectations; a “sampling” of neuromodulation may be a useful “taster,” but is unlikely to allow most residents to achieve proficiency unless they set aside elective time for the endeavor.

Based on the current state of the field, the requirement of a fellowship may be premature but is certainly looming. Numerous fellowships now exist around the country. Most are focused on interventional psychiatry and thus limited to psychiatrists; however, an intriguing development has been the creation of cross-disciplinary “neuromodulation” fellowships open to physicians from diverse clinical training backgrounds including psychiatry, neurosurgery, neurology, and physical medicine and rehabilitation (PM&R) (23). This cross-disciplinary training approach may have some advantages, such as promoting new ideas and applications for available neurotechnologies or fostering multidisciplinary research and clinical collaborations. Indeed, cross-disciplinary fellowship training mirrors a trend in subspecialties such as interventional neuroradiology/endovascular surgery, where clinicians from various training backgrounds can receive fellowship training in a common field of interest (24, 25). These parallel procedural, high-tech subspecialty training programs can serve as templates for how to consider restructuring interventional psychiatry training over time. The necessity of formalized fellowship training in interventional psychiatry may be dependent on the evolving scope of practice within this field, discussed below.

## What is an Interventional Psychiatrist's Scope of Practice?

The short answer to this question is “whatever they were trained and/or credentialed to do.” Common examples include the administration of ECT, TMS, or ketamine/esketamine, the periprocedural evaluation and management of patients with treatment refractory psychiatric illnesses, and the programming and management of DBS and VNS devices for psychiatric indications. However, significant speculation toward the future can be offered. Often procedural specialties have an evolving scope of practice based on new technologies and new clinical needs. For example, the development of novel endovascular neurosurgical tools and techniques for aneurysm coiling, carotid artery stenting, and clot retrieval has drawn representatives from multiple specialties, including traditionally non-surgical specialties such as neurology and radiology, to seek interventional fellowship training, especially as the clinical demand and infrastructure has grown. Likewise, pain management clinicians with backgrounds in anesthesiology, neurology, PM&R, emergency medicine, and psychiatry have trained in invasive neuromodulation techniques to manage pain conditions. Scope of practice is thus adapted through the vehicle of fellowship training, expanded residency training, or medical education workshops to teach clinicians to master and incorporate new technologies and skills that are not customarily part of any specific residency training program. In the pain management example above, a board-certified psychiatrist can therefore complete a pain medicine fellowship to themselves implant spinal cord neurostimulators for pain, subsequently deemed within their scope of practice.

Interventional psychiatrists similarly need to remain poised to adopt, master and incorporate new technologies and treatment modalities as they arise. Potential areas of future practice expansion include incorporation of new non-invasive technologies such as MST, transcranial electrical stimulation, focused ultrasound and trigeminal nerve stimulation. Implantation and management of both emerging and established invasive technologies for psychiatric disease may also be within the scope of interventional psychiatry practice in the future. This could include implanting VNS, DBS, responsive neurostimulation devices, or epidural cortical stimulation devices for depression and other neuropsychiatric conditions. Traditionally, neurosurgeons have performed the more invasive neuromodulation procedures due to the higher degree of skill required and the higher safety risk, thus the necessity of a steady caseload to keep up one's technique. However, as these technologies obtain new indications for highly prevalent psychiatric conditions such as major depressive disorder (lifetime prevalence 20.6%) (26) and obsessive compulsive disorder (lifetime prevalence 2.3%) (27), there will likely be increased demand for these neuromodulation procedures and well-trained clinicians to provide them. Interventional modalities often have superior safety and cost-effectiveness profiles (28–34) in treating medication-refractory patients, leading clinicians to call for their use earlier in a disease course (35, 36) and further increasing clinical demand.

Current interventional psychiatry fellowship does not train psychiatrists in stereotactic and functional neurosurgical techniques such as device implantation. Were interventional psychiatrists motivated to pursue additional training in these invasive, higher-risk procedures, hybrid invasive/non-invasive training programs could emerge. At that stage, interventional psychiatry fellowship training would become a necessity likely requiring two or more years duration, akin to interventional neurology fellowships designed to teach neurologists to perform intravascular procedures. This longer training would ensure adequate procedural skill for achieving successful and safe patient outcomes.

Based on the current state of the field and the rapid advancement of treatment techniques, we recommend that, at a minimum, interventional psychiatry practitioners complete a 1-month interventional psychiatry rotation during residency training or hands-on continuing education training course in the modality of interest prior to its implementation and use. Details of minimum number of treatments to achieve proficiency, regulatory bodies to enforce requirements, grandfathering of current practitioners, and continuing medical education requirements within the expanding subspecialty are all important topics for future discussion. An outline of potential competency areas for a psychiatric neuromodulation or interventional psychiatry fellowship is provided in Table 1.

## Discussion

Neuromodulation is a discipline focused on the application of various technologies to the nervous system to effect therapeutic change. It has clinical utility in several branches of medicine including psychiatry, neurology, neurosurgery, pain management, and PM&R. Interventional psychiatry is the subspecialty “home” for providers with experience in psychiatric neuromodulation or other procedural techniques. As has been the case for neuromodulation subspecialties in other areas of medicine, interventional psychiatry is rapidly evolving, both in terms of scope of practice and training needs. This article addresses some important looming questions for the field to consider as leaders and educators contemplate how to adequately train and define the role of interventional psychiatrists. Interventional psychiatry is one of the most exciting, rapidly growing, and promising fields of medicine; responsibly ushering in new therapeutics to providers with the clinical acumen and skill to administer them safely and effectively is a critical step toward improving patient health, function, and well-being.

## Author Contributions

NT was involved in the conceptualization of the work and writing the original draft of the manuscript. NW was involved in conceptualization and editing of the manuscript. Both authors contributed to the article and approved the submitted version.

## Conflict of Interest

The authors declare that the research was conducted in the absence of any commercial or financial relationships that could be construed as a potential conflict of interest.

## Publisher's Note

All claims expressed in this article are solely those of the authors and do not necessarily represent those of their affiliated organizations, or those of the publisher, the editors and the reviewers. Any product that may be evaluated in this article, or claim that may be made by its manufacturer, is not guaranteed or endorsed by the publisher.
